# Characterization of pediatric patients receiving erythropoiesis-stimulating agents for management of anemia of chronic kidney disease in the UK and US: a feasibility assessment using retrospective chart review

**DOI:** 10.1186/s12882-026-05188-2

**Published:** 2026-08-27

**Authors:** Sally Mountcastle, Keele Wurst, Alice Rouleau, Neil R. Brett, Jennifer McKenzie, Justyna Amelio, Kathy Fraeman, Jake Hunnicutt, Susanna Cuadripani, David Webb, Amrit Kaur, Rukshana Shroff, Eleonora Staines Urias, Anna Richards, Thomas F. Hiemstra

**Affiliations:** 1 GSK, Collegeville, PA USA; 2 PPD, Part of Thermo Fisher Scientific, Paris, France; 3 PPD, Part of Thermo Fisher Scientific, Montreal, Quebec Canada; 4 GSK, Stevenage, Hertfordshire UK; 5 PPD, Part of Thermo Fisher Scientific, Bethesda, MD USA; 6 GSK, 79 New Oxford Street, London, WC1A 1DG UK; 7 Manchester University NHS Foundation Trust, Manchester, UK; 8 UCL Great Ormond Street Institute of Child Health, London, UK

**Keywords:** Anemia of chronic kidney disease, Clinical management, Erythropoiesis-stimulating agents, Pediatric patients

## Abstract

**Background:**

Anemia is a common complication of chronic kidney disease (CKD). We evaluated the feasibility of collecting comprehensive data from medical charts for longitudinal assessment of anemia among pediatric patients receiving erythropoiesis-stimulating agents (ESAs) for anemia of CKD in the United Kingdom (UK) and United States (US).

**Methods:**

This retrospective chart review study used an electronic case report form to collect data from medical records of pediatric patients treated with an ESA (as either an incident or a prevalent patient) from January 1, 2017–December 31, 2018. Consecutive chart reviews were conducted from November 1–December 20, 2021. Data collected included clinical characteristics, disease, and treatment management.

**Results:**

Of 50 patients enrolled, 36 (72.0%) and 14 (28.0%) were from the UK and US, respectively; median (interquartile range) age was 9.0 (4.0–11.0) years, 22 (44.0%) were female and 28 (56.0%) were male. Median follow-up was 52.1 weeks. The most common primary CKD etiology was congenital anomalies of the kidney/urinary tract (*n* = 14, 28.0%). Twenty-three (46.0%) patients received dialysis at the index date and eight (16.0%) had a kidney transplant post-index date. Twenty-eight (56.0%) patients received iron treatment with their index-line ESA. The mean (standard deviation) baseline hemoglobin level was 10.0 (2.0) g/dL. Twenty adverse events (AEs) were recorded in nine (18.0%) patients; all were serious and no inference was sought for attributability to treatment.

**Conclusions:**

This study demonstrates feasibility of retrospectively extracting clinical characteristics, treatment history, and AEs from medical charts of pediatric patients with anemia of CKD.

**Supplementary information:**

The online version contains supplementary material available at https://doi.org/10.1186/s12882-026-05188-2.

## Introduction

Pediatric chronic kidney disease (CKD) is relatively uncommon, with an estimated global prevalence of 15–74.7 per million children [1–4]. Anemia is a challenging complication of CKD, occurring in approximately 73–93% of pediatric patients with CKD stage 3–5, with worsening severity in more advanced stages [5–7]. Anemia of CKD in children is associated with impaired daily functioning, reduced quality of life, increased risk of all-cause hospitalization, poor cardiovascular outcomes, and increased morbidity and mortality [5, 8–10].

Erythropoiesis-stimulating agents (ESAs) and iron therapies represent the foundation for managing anemia of CKD [6, 11]. According to the United States Renal Data System, ESAs are prescribed to 34.9% of children prior to initiating end-stage kidney disease (ESKD) therapy [12]. Although patients with anemia of CKD can be managed with ESAs, hyporesponsiveness can occur. This may be caused by iron deficiency, inflammation and hepcidin accumulation, uremia, vitamin deficiency, and inadequate dialysis [13]. ESA hyporesponsiveness is mostly reported in adults with anemia of CKD [5], and is associated with an increased risk of cardiovascular hospitalization and higher treatment costs among patients undergoing hemodialysis (HD) [13–15]. Peritoneal dialysis (PD) is usually preferred and recommended over HD in pediatric patients [16, 17] due to the difficulty in maintaining a vascular access route for HD and having a more limited impact on normal activities and education [18].

The treatment landscape of anemia of CKD is still evolving; therapies such as hypoxia-inducible factor prolyl hydroxylase inhibitors (HIF-PHIs) have received marketing authorization as oral treatments for anemia in adult patients receiving dialysis in the United States (US), and in adult patients in Japan and Europe [19–22]. Clinical development research for HIF-PHIs in pediatric populations is ongoing (NCT05970172; registration date: July 25, 2023), with the ASCEND-P trial (NCT05682326; registration date: January 4, 2023) having been discontinued.

Observational, registry-based studies are important to understanding the current care and management of patients, as well as helping to inform clinical practice [8]. For example, a retrospective observational study of adult patients with non-dialysis-dependent CKD found that monitoring of hemoglobin and iron deficiencies following initiation of ESA treatment were suboptimal, highlighting a need for more consistent follow-up of patients [23]. However, the current understanding of the clinical management of children with anemia of CKD is limited to a few studies with small sample sizes [6]. We conducted an exploratory, retrospective medical chart review of 50 pediatric patients (aged < 18 years) receiving ESAs for anemia of CKD in the United Kingdom (UK) and US. The aims of this study were to extract data by medical chart review and to assess the completeness of these data for descriptions of anemia treatment, hemoglobin changes, and adverse events (AEs) in this population. The feasibility of using medical chart review data to inform the design of an external control arm for a single-arm clinical trial and refine data collection for future real-world studies was evaluated.

### Objectives

The objective of the study was to assess the feasibility of extracting data by using medical chart review to describe pediatric patients with anemia of CKD treated with ESAs in terms of their:

1. Clinical characteristics and treatment management.
2. Availability of hemoglobin data during treatment with ESAs.
3. AEs recorded as occurring during ESA treatment.

The availability, quality and completeness of data in each of these areas were assessed.

## Methods

### Study design and data collection

**Fig. 1:**
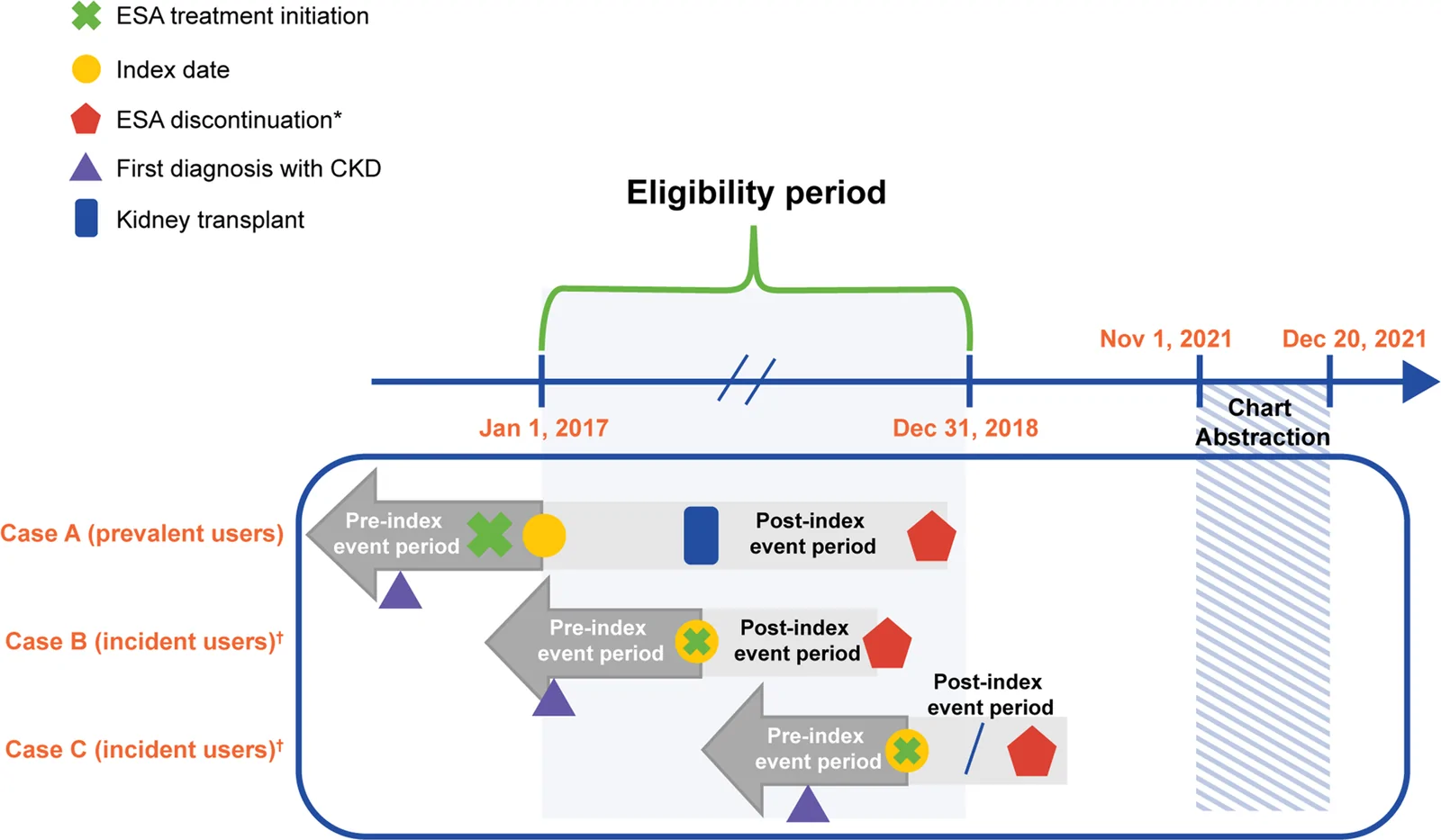
Study schematic. *or 52 weeks after receipt of a kidney transplant for transplant recipients, loss to follow-up, end of follow-up, or death, whichever occurred first. ^†^For the purpose of the study, patients who started ESA therapy within the eligibility period were defined as incident patients. However, these patients may have received ESA therapy over the duration of their CKD history. CKD, chronic kidney disease; ESA, erythropoiesis-stimulating agent

Retrospective consecutive chart reviews were conducted to evaluate the characteristics and treatment of 50 pediatric patients with anemia of CKD in the UK and US (Fig. 1). Reviews were conducted by treating physicians or authorized delegated staff at three centers in the UK, and by treating physicians only in the US. Physicians (or delegated staff in the UK) were asked to include eligible patients, prioritizing those with a recent medical visit to decrease the possibility of selection bias, until the target sample size was achieved (overall, *N* = 50). At study outset, it was also predetermined that a recruitment split target of approximately 40% of patients enrolled would be receiving dialysis (with 60% not receiving dialysis). This target was deemed the best way to provide a breadth of information, while suitably representing treatment practice and evolution across CKD stages 3–5 (including PD and HD) patients within the planned sample.

The index date was defined as the date a patient started ESA therapy within the eligibility period (January 1, 2017–December 31, 2018). For patients who initiated ESA therapy prior to the eligibility period (prevalent users), January 1, 2017 was used as their index date (Fig. 1). The first date of ESA initiation was used as the index date for patients with ≥1 record(s) of ESA exposure (i.e., patients switching ESAs) during the eligibility period. Index-line ESA refers to the type of ESA treatment initiated or ongoing for each patient at their index date. Data for patients with an index date for ESA prescription during the eligibility period were collected via patient-level electronic case report forms (eCRFs). For the purpose of the study, patients were defined as being an incident (newly commenced) or a prevalent (established) ESA user during the eligibility period (Fig. 1). Incident ESA users were not necessarily treatment-naïve and may have had a period of ESA treatment prior to the eligibility period, whereas prevalent users were those receiving an ESA as the eligibility period commenced.

The study period for each patient constituted a pre-index period (any time prior to the index date available in the medical chart) and post-index period (until permanent ESA discontinuation or up to 52 weeks from the index date, loss to follow-up, or death; Fig. 1). Patients receiving a kidney transplant during the post-index period were subsequently followed as part of a secondary cohort for up to 52 weeks post-transplant or until permanent ESA discontinuation, loss to follow-up, or death, whichever occurred first after the transplant. Patient follow-up and data extraction was continued in the post-transplant period to assess how anemia parameters and treatment practices changed in this setting. AEs recorded in the post-index period were captured as free text and coded using the Medical Dictionary for Regulatory Activities; no inference was sought for attributability to ESA treatment. Data quality for all objectives was assessed by cleaning (removal of incorrect or poorly formatted data) and monitoring (combination of automatic edit checks, manual checks, and clinical review). Data collection, including clinical characteristics, disease, and treatment management parameters, was conducted from November 1–December 20, 2021.

### Ethical approval

The study protocol was reviewed and approved by institutional review boards (Advarra, Columbia, Maryland, US and the Health Research Authority, UK), in accordance with the International Council on Harmonisation of Technical Requirements for Registration of Pharmaceuticals for Human Use (ICH) Good Clinical Practice (GCP) and applicable country-specific requirements. Moreover, the study was performed in accordance with ICH GCP, and all applicable participant privacy requirements and principles outlined in the Declaration of Helsinki. Informed consent waivers were approved by Advarra (US) and the Health Research Authority (UK) due to the retrospective study design and the pseudonymized nature of the data. All data collected in this study were treated as strictly confidential in accordance with country-specific regulations. Identifying information, for example, patient initials or date of birth were not entered into the eCRF.

### Patients

Patients from the UK and US were included if they met the following inclusion criteria: age < 18 years at the index date; CKD of any cause; and treatment with an ESA during the eligibility period. CKD was defined as stage 3–5 disease with an eGFR < 60 mL/min/1.73 m^2^ persistence for ≥3 months. Dialysis could be classified as receipt of PD every 1–2 days or receipt of HD ≥ 3 times per week. Patients who had undergone kidney transplantation before the eligibility period were eligible for inclusion, provided they had CKD and were receiving an ESA. Key exclusion criteria included: a history of malignancy within 2 years prior to the index date; receipt of cancer therapy in the post-index period; anemia due to causes other than CKD; and participation in another CKD or anemia trial in the post-index period.

### Statistical analyses

The sample size of 50 patients was not formally calculated but was selected based on overall study conduct feasibility and recruitment assessment feedback. Descriptive statistical analyses were conducted. The Kaplan–Meier estimator was used to analyze time-to-event outcomes, reporting cumulative incidence or survival times, as appropriate, with corresponding 95% confidence intervals (CIs). Continuous variables were summarized as mean (standard deviation [SD]) or median (range/interquartile range [IQR]) as appropriate. Standardized ESA doses were calculated by conversion to the equivalent dose of IV epoetin. Post-hoc analyses were performed to understand the feasibility of adding country-level subgroup analyses for key outcomes across the study objectives, and to assess additional outcomes for Objective 2, i.e., the number and frequency of hemoglobin measurements available during follow-up. AEs were captured from the retrospective chart review; there was no inference with respect to attribution of any AEs with ESAs. Statistical analyses were performed in SAS EG version 7.1 or higher.

## Results

### Feasibility assessment: data availability

It was feasible to efficiently extract data using retrospective chart review. The requested data were sufficiently clean, reasonably complete, and reliable at the index date and throughout follow-up to meet the study objectives. Date of initial CKD and kidney anemia diagnoses were missing for seven (14.0%) and 14 (28.0%) patient charts during the pre-index period, respectively (Supplementary Table 1). The following information was missing from patient charts on the index date: CKD stage (*n* = 1; 2.0%), eGFR (*n* = 15; 30.0% [noting an eGFR measurement was not applicable in patients on dialysis but was a prespecified measure in the eCRF]), hemoglobin concentration (*n* = 1; 2.0%; Supplementary Table 1), and date of hemoglobin measurement (*n* = 8; 16.0%). Data availability differed for UK and US patients in this study; fewer patients in the UK had missing data for date of kidney anemia diagnosis (16.7% vs. 57.1%), eGFR (19.4% vs. 57.1%), and date of hemoglobin measurement (2.8% vs. 50.0%) than for the US (Supplementary Table 2).

### Patients

A total of 50 patients were screened and enrolled in the study: 36 (72.0%) patients were from the UK and 14 (28.0%) patients from the US. Overall, the median (IQR) age was 9.0 (4.0–11.0) years, 44.0% of patients were female and 56.0% were male. The mean (SD) weight (*n* = 41) and height (*n* = 40) was 24.8 (13.1) kg and 114.5 (27.4) cm, respectively at the index date. At the index date, 27 (54.0%) patients were incident ESA users and 23 (46.0%) patients were prevalent ESA users.

**Patient characteristics:** **UK vs. US patients**

Data were extracted for 36 patients across three participating UK centers and 14 patients from two US physicians. All prevalent ESA users were from the UK (63.9% of all UK patients) (Table 1). All UK patients were treated at public hospitals while US patients were treated at private hospitals (*n* = 6), private offices (*n* = 4), or public hospitals (*n* = 4). Baseline CKD stage information was available for 49 patients; 17 (34.7%) patients had ESKD while the remaining patients had CKD stage 3–5.

**Table 1:** Characteristics of UK and US patients

Characteristic, n (%)*		Country	
	All (N = 50)	UK (n = 36)	US (n = 14)
Age, median (IQR) years	9.0 (4.0–11.0)	7.0 (2.0–10.5)	10.5 (9.0–13.0)
Sex			
Male Female	28 (56.0) 22 (44.0)	21 (58.3) 15 (41.7)	7 (50.0) 7 (50.0)
Received dialysis at index date Hemodialysis Peritoneal dialysis Did not receive dialysis at index date	23 (46.0) 15 (30.0) 8 (16.0) 27 (54.0)	16 (44.4) 10 (27.8) 6 (16.7) 20 (55.6)	7 (50.0) 5 (35.7) 2 (14.3) 7 (50.0)
Age of patients receiving dialysis at index, median (IQR) years			
Hemodialysis Peritoneal dialysis	9.0 (3.0–11.0) 11.0 (5.5–13.5)	7.5 (1.0–9.0) 11.0 (2.0–13.0)	9.0 (9.0–11.0) 12.0 (9.0–15.0)
CKD stage (*n* = 49) 3 4 5 ESKD	9 (18.4) 11 (22.4) 12 (24.5) 17 (34.7)	5 (14.3) 9 (25.7) 10 (28.6) 11 (31.4)	4 (28.6) 2 (14.3) 2 (14.3) 6 (42.9)
Type of ESA user at index date			
Incident^†^ Prevalent^‡^	27 (54.0) 23 (46.0)	13 (36.1) 23 (63.9)	14 (100) 0
ESA line at the index date			
1 2	40 (80.0) 10 (20.0)	26 (72.2) 10 (27.8)	14 (100) 0
ESA lines received in the post-index period			
1 2	46 (92.0) 4 (8.0)	33 (91.7) 3 (8.3)	13 (92.9) 1 (7.1)

*Unless otherwise specified

^†^Incident users initiated ESA during the eligibility period

^‡^Prevalent users initiated ESA prior to the eligibility period

CKD, chronic kidney disease; ESA, erythropoiesis-stimulating agent; ESKD, end-stage kidney disease; IQR, interquartile range; UK, United Kingdom; US, United States

Most patients in the overall population (*n* = 40; 80.0%) were receiving their first ESA treatment line at their index date; the remaining patients (*n* = 10; 20.0%) had been switched to a second line of ESA treatment. In the overall population, 46 (92.0%) patients received one line of ESA therapy post-index date (33 [91.7%] patients in the UK; 13 [92.9%] patients in the US). The remaining patients received two lines of ESA therapy post-index date. Similar proportions of patients by age received HD and PD in the UK and US.

**Patient characteristics:** **patients receiving/not receiving dialysis**

A total of 23 (46.0%) patients received dialysis at their index date; 15 (30.0%) received HD and eight (16.0%) received PD (Table 2).

**Table 2:** Characteristics of patients by dialysis status at index date

Characteristic, n (%)*		Dialysis status at index date	
	All (N = 50)	Not on dialysis (n = 27)	On dialysis (n = 23)
Age, median (IQR) years	9.0 (4.0–11.0)	8.0 (4.0–11.0)	9.0 (3.0–13.0)
Sex			
Male Female	28 (56.0) 22 (44.0)	18 (66.7) 9 (33.3)	10 (43.5) 13 (56.5)
BMI, mean (SD) kg/m^2^	17.5 (3.0)	17.6 (2.7)	17.5 (3.7)
Height, mean (SD) cm Missing	114.5 (27.4) 10 (20.0)	112.8 (23.4) 2 (7.4)	117.3 (33.6) 8 (34.8)
Weight, mean (SD) kg Missing	24.8 (13.1) 9 (18.0)	23.6 (11.1) 1 (3.7)	26.9 (16.2) 8 (34.8)
Lab measurements, mean (SD) baseline			
eGFR, mL/min/1.73 m^2^			
Patients	35 (70.0)	23 (85.2)	12 (52.2)
Mean (SD)	15.2 (9.1)	15.6 (9.9)	14.3 (7.8)
Missing	15 (30.0)	4 (14.8)	11 (47.8)
Hemoglobin (g/dL)			
Patients	49 (98.0)	26 (96.3)	23 (100.0)
Mean (SD)	10.0 (2.0)	10.5 (1.8)	9.5 (2.0)
Missing	1 (2.0)	1 (3.7)	0
PTH, pg/mL			
Patients with change in PTH value during index-line treatment	18 (36.0)	8 (29.6)	10 (43.5)
Patients	10 (55.6)	4 (50.0)	6 (60.0)
Median (IQR)	193.3 (76.0–976.0)	193.3 (99.5–493.3)	544.5 (76.0–1567.0)
Missing	8 (44.4)	4 (50.0)	4 (40.0)
Dialysis type			
Hemodialysis Peritoneal dialysis	15 (30.0) 8 (16.0)	– –	15 (65.2) 8 (34.8)
Time since starting dialysis, mean (SD) months	9.4 (20.2)	–	9.4 (20.2)
Primary CKD etiology			
Congenital anomalies Combined rare diseases Obstructive nephropathy Nephronophthisis Other causes Glomerulopathy Unknown	14 (28.0) 11 (22.0) 7 (14.0) 6 (12.0) 6 (12.0) 5 (10.0) 3 (6.0)	8 (29.6) 6 (22.2) 5 (18.5) 3 (11.1) 4 (14.8) 0 2 (7.4)	6 (26.1) 5 (21.7) 2 (8.7) 3 (13.0) 2 (8.7) 5 (21.7) 1 (4.3)
CKD stage (*n* = 49)			
3 4 5 ESKD^†^	9 (18.4) 11 (22.4) 12 (24.5) 17 (34.7)	9 (34.6) 9 (34.6) 5 (19.2) 3 (11.5)	0 2 (8.7) 7 (30.4) 14 (60.9)
Time since diagnosis, mean (SD) months			
CKD (*n* = 43) Kidney anemia^‡^ (*n* = 36)	43.3 (48.9) 22.9 (34.7)	44.6 (47.6) 25.1 (37.0)	41.4 (52.2) 19.0 (31.1)

*Unless otherwise specified; categories with low numbers of patients were excluded to maintain anonymity

^†^ESKD staging allocation was different to the HD/PD group total of 23

^‡^Kidney anemia diagnosis was determined by individual physicians

BMI, body mass index; CKD, chronic kidney disease; eGFR, estimated glomerular filtration rate; ESKD, end-stage kidney disease; HD, hemodialysis; IQR, interquartile range; PD, peritoneal dialysis; PTH, parathyroid hormone

At index date, the mean (SD) baseline eGFR was 15.6 (9.9) mL/min/1.73 m^2^ for patients not receiving dialysis. The mean (SD) baseline hemoglobin level in the overall population was 10.0 (2.0) g/dL; the difference between patients receiving and not receiving dialysis was negligible. Overall, 18 patients (36.0%) had a change in parathyroid hormone (PTH) levels during index-line ESA treatment (not on dialysis, *n* = 8 [29.6%]; on dialysis, *n* = 10 [43.5%]). Of those, there was a substantial level of data missingness when assessing PTH level changes (pg/mL) during index-line treatment (overall population, *n* = 8 [44.4%]; not on dialysis, *n* = 4 [50.0%]; on dialysis, *n* = 4 [40.0%]). The overall (*n* = 10) median (IQR) PTH value for patients with a change during index-line was 193.3 (76.0–976.0) pg/mL (not on dialysis, *n* = 4, 193.3 [99.5–493.3] pg/mL; on dialysis, *n* = 6, 544.5 [76.0–1567.0] pg/mL).

The most common primary CKD etiologies were congenital anomalies (*n* = 14; 28.0%), obstructive nephropathy (*n* = 7; 14.0%), and nephronophthisis (*n* = 6; 12.0%). CKD etiology was pooled for 11 patients with rare conditions related to anemia of CKD and not granularly reported to maintain patient anonymity. Generally, CKD etiology did not differ notably for patients who received dialysis vs. those who did not. However, obstructive nephropathy was more frequent amongst those not on dialysis than those on dialysis (18.5% vs. 8.7%); glomerulopathy was a more frequent etiology among patients on dialysis than those not on dialysis (21.7% vs. 0.0%) (Table 2).

The mean (SD) time since diagnosis was 43.3 (48.9) months for CKD and 22.9 (34.7) months for kidney anemia in patients with available data. The most common comorbidities in the overall population were hypertension (*n* = 15; 30.0%), neurocognitive impairment (*n* = 11; 22.0%), and growth abnormalities (*n* = 10; 20.0%) (Supplementary Table 3). Overall, two patients (4.0%) were described as having chronic inflammation and one (2.0%) was described as having acute inflammation. A total of 13 (26.0%) patients were listed as having rare comorbidities, but these were not reported individually to maintain the anonymity of patients.

Median (95% CI) time to first kidney transplant from index date was 37.07 (23.1–43.1) weeks (Supplementary Fig. 1). The median (IQR) length of follow-up was 52.1 (52.1–52.1) weeks in the overall population. The median (IQR) follow-up was longer for patients who received a kidney transplant during the eligibility period (*n* = 8; 59.1 [41.1–80.0] weeks) than those with no transplant (*n* = 42; 52.1 [52.1–52.1] weeks). During follow-up, dialysis status changed for 5/23 (21.7%) patients (three PD patients changed to receive HD; one HD patient changed to less frequent dialysis; one HD patient stopped dialysis) and eight (16.0%) patients had a kidney transplant. The CKD stages of patients with a transplant in the post-index period were as follows: stage 3, *n* = 1/8 (12.5%); stage 4, *n* = 2/8 (25.0%); stage 5, *n* = 4/8 (50.0%); ESKD, *n* = 1/8 (12.5%). By the end of follow up period, only 1/8 (12.5%) had stage 3 and 2/8 (25.0%) patients had ESKD. Overall, four (8.0%) patients had received a kidney transplant prior to the index date (not on dialysis, *n* = 1/27 [3.7%]; on dialysis, *n* = 3/23 [13.0%]). Data on time-from-kidney transplantation until index date were available for these four patients (mean [SD] time, 42.3 [36.1] months).

### Anemia treatment

**Fig. 2:**
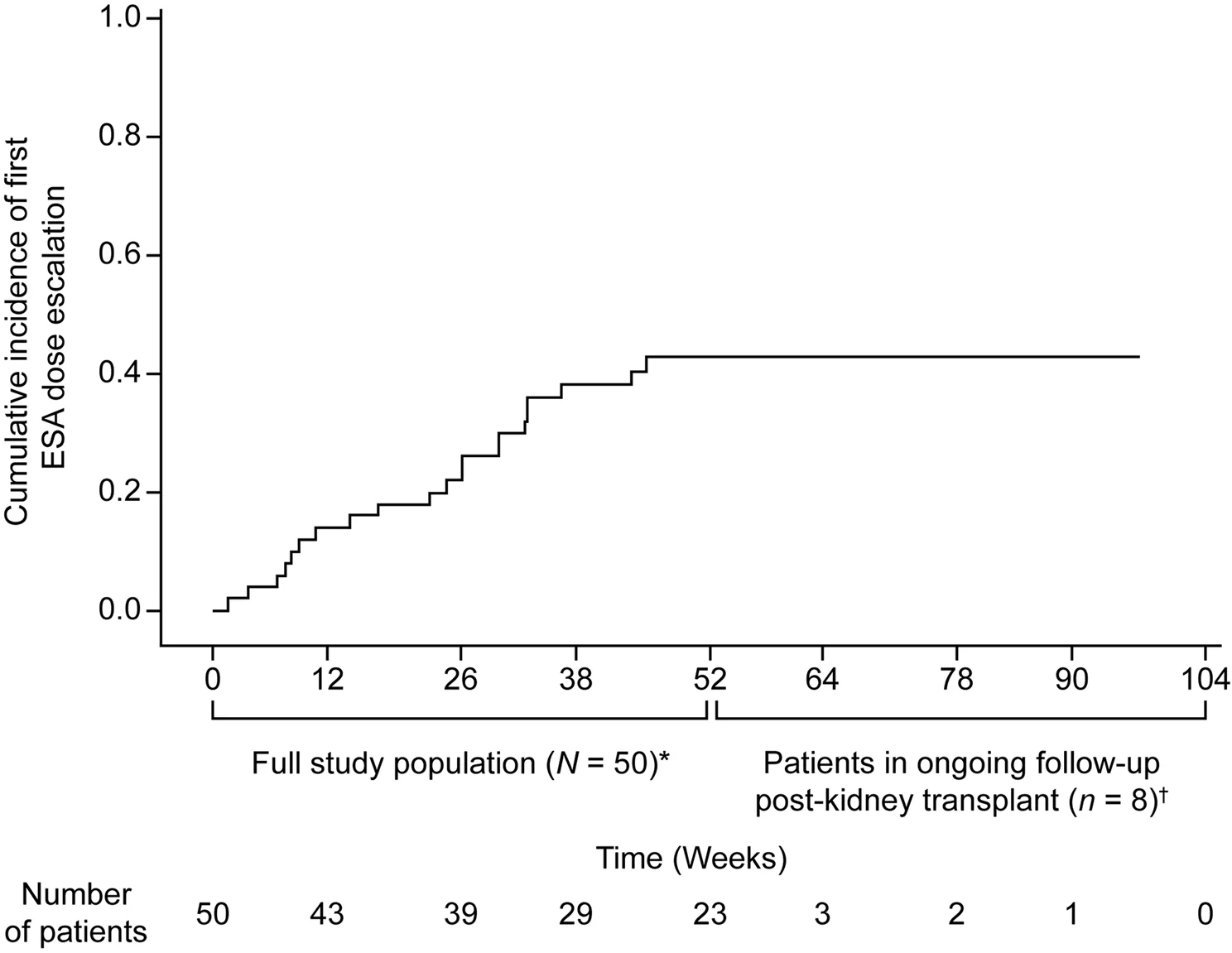
Time from index date to first ESA dose escalation in the overall population. (N = 50). *Including patients who had a kidney transplant. ^†^Patients who had a kidney transplant during the study period were followed for a further 52 weeks from their date of transplant. The number of patients from Week 52–104 decreased as patients reached their end of follow-up. ESA; erythropoiesis-stimulating agent

The most common type of ESA used at index date was darbepoetin alfa (*n* = 27 [54.0%]). Other types of ESA received included erythropoietin (*n* = 10 [20.0%]), epoetin alfa (*n* = 7 [14.0%]) and epoetin beta (*n* = 6 [12.0%]) (Table 3). A total of seven (14.0%) patients discontinued their index-line ESA at or before the end of follow-up; reasons for discontinuation included condition improved/maintained, patient decision, loss to follow-up, and death (all *n* = 1; Table 3). The median (IQR) time to index-line ESA discontinuation was 10.5 (9.0–34.0) months in the overall population and was longer for patients on dialysis (38.0 [34.0–42.0] months) than not on dialysis (9.0 [8.5–10.5] months). Four (8.0%) patients received a subsequent line of ESA after their index-line, all of whom were on dialysis (Table 3). Subsequent treatments included epoetin alfa (*n* = 2), darbepoetin alfa, and peginesatide (both *n* = 1). The cumulative incidence curve for time from index date to the first ESA dose escalation is shown in Fig. 2; by Week 52, 27 patients had an increase in ESA dose.

**Table 3:** Anemia treatment received during the pre-index period and at the index date by dialysis status

Characteristic, n (%)*		Dialysis status at index date	
	All (N = 50)	Not on dialysis (n = 27)	On dialysis (n = 23)
Incident ESA users	27 (54.0)	15 (55.6)	12 (52.2)
No ESA prior to index-line	25 (92.6)	13 (86.7)	12 (100)
≥1 ESA prior to index-line	2 (7.4)	2 (13.3)	0
Prevalent ESA users	23 (46.0)	12 (44.4)	11 (47.8)
No ESA prior to index-line	15 (65.2)	8 (66.7)	7 (63.6)
≥1 ESA prior to index-line	8 (34.8)	4 (33.3)	4 (36.4)
Type of ESA used at index date^†^			
Darbepoetin alfa	27 (54.0)	15 (55.6)	12 (52.2)
Erythropoietin	10 (20.0)	4 (14.8)	6 (26.1)
45 μg/kg	1 (10.0)	0	1 (16.7)
100 units/kg	1 (10.0)	0	1 (16.7)
50 μg	1 (10.0)	0	1 (16.7)
75 μg	1 (10.0)	0	1 (16.7)
8 mg/kg	3 (30.0)	3 (75.0)	0
Other	2 (20.0)	1 (25.0)	1 (16.7)
Missing	1 (10.0)	0	1 (16.7)
Epoetin alfa	7 (14.0)	4 (14.8)	3 (13.0)
0.75 μg/kg	1 (14.3)	1 (25.0)	0
50 units/kg	4 (57.1)	3 (75.0)	1 (33.3)
50 μg	1 (14.3)	0	1 (33.3)
Other	1 (14.3)	0	1 (33.3)
Epoetin beta	6 (12.0)	4 (14.8)	2 (8.7)
Other	5 (83.3)	4 (100)	1 (50.0)
75 μg	1 (16.7)	0	1 (50.0)
ESA administration at index date			
Intravenous Subcutaneous	18 (36.0) 32 (64.0)	7 (25.9) 20 (74.1)	11 (47.8) 12 (52.2)
Discontinued index-line ESA at or before the end of follow-up	7 (14.0)	4 (14.8)	3 (13.0)
Reasons for discontinuing index-line ESA^‡^			
Condition improved/maintained Patient decision Loss to follow-up Death Other Missing	1 (14.3) 1 (14.3) 1 (14.3) 1 (14.3) 1 (14.3) 2 (28.6)	1 (25.0) 0 1 (25.0) 1 (25.0) 1 (25.0) 0	0 1 (33.3) 0 0 0 2 (66.7)
Median (IQR) time to index-line ESA discontinuation, months^§^	10.5 (9.0–34.0)	9.0 (8.5–10.5)	38.0 (34.0–42.0)
Received a subsequent line of ESA after the index-line	4 (8.0)	0	4 (17.4)
Type of ESA received after the index-line			
Epoetin alfa Darbepoetin alfa Peginesatide	2 (50.0) 1 (25.0) 1 (25.0)	0 0 0	2 (50.0) 1 (25.0) 1 (25.0)
Received iron treatment at index date or any time prior to index date	33 (66.0)	18 (66.7)	15 (65.2)
Receiving iron treatment during index-line ESA Oral^§^ Intravenous^§^	28 (56.0) 18 (64.3) 10 (35.7)	15 (55.6) 13 (86.7) 2 (13.3)	13 (56.5) 5 (38.5) 8 (61.5)
Patients receiving red blood cell transfusion between index date and end of follow-up	3 (6.0)	0	3 (13.0)
Had a kidney transplant in the post-index period	8 (16.0)	7 (19.4)	1 (7.1)

*Unless otherwise specified

^†^Excluded ‘missing’ in the denominator

^‡^Percentages calculated as a percentage of the number of patients who discontinued their index-line ESA at or before the end of follow-up (*n* = 7 for the overall population; *n* = 4 for patients not on dialysis; *n* = 3 for patients on dialysis)

^§^Percentages based on proportion of patients (overall or by non-dialysis and dialysis status) receiving iron treatment during index-line ESA

ESA, erythropoiesis-stimulating agent; IQR, interquartile range

The index-line ESAs received by patients who underwent kidney transplant in the post-index period (*n* = 8; 16.0%) were darbepoetin alfa (*n* = 6), erythropoietin, and epoetin beta (*n* = 1 each). Four transplant recipients discontinued their index-line ESA on or before the end of follow-up; documented reasons included condition improved/maintained and other (*n* = 1 each). Reasons for discontinuation were missing for two transplant recipients. The median (IQR) time to index-line ESA discontinuation among transplant recipients was 21.5 (9.0–38.0) months.

A total of 33 (66.0%) patients received iron therapy prior to or at their index date, and this number was consistent across dialysis subgroups (Table 3). Fewer patients (*n* = 28; 56.0%) received iron treatment during their index-line ESA, and only one (3.6%) of these patients started a new iron therapy during their index-line ESA. Of the 28 patients who received iron therapy during their index-line ESA, 18 (64.3%) received oral treatment and 10 (35.7%) received IV treatment.

### Frequency of hemoglobin testing during ESA treatment

The median (IQR) number of times that serum hemoglobin was tested during receipt of the index-line ESA was 11.0 (3.0–22.0) measurements. The frequency of hemoglobin testing showed some variation, with a higher average number of tests recorded for patients on dialysis (median [IQR] 20.0 [3.0–50.0]) than those not on dialysis (6.0 [2.0–16.0]]. Testing was also more frequent for patients who received a kidney transplant in the post-index period (median [IQR], 40.0 [9.5–87.5]) than those who did not (8.0 [3.0–22.0]), and for patients in the UK (19.5 [8.0–27.0]) than those in the US (2.0 [2.0–3.0]). Hemoglobin levels were assessed less often during the subsequent line of ESA therapy, with a median (IQR) of 4.0 (1.5–13.5) times in the overall population.

**Fig. 3:**
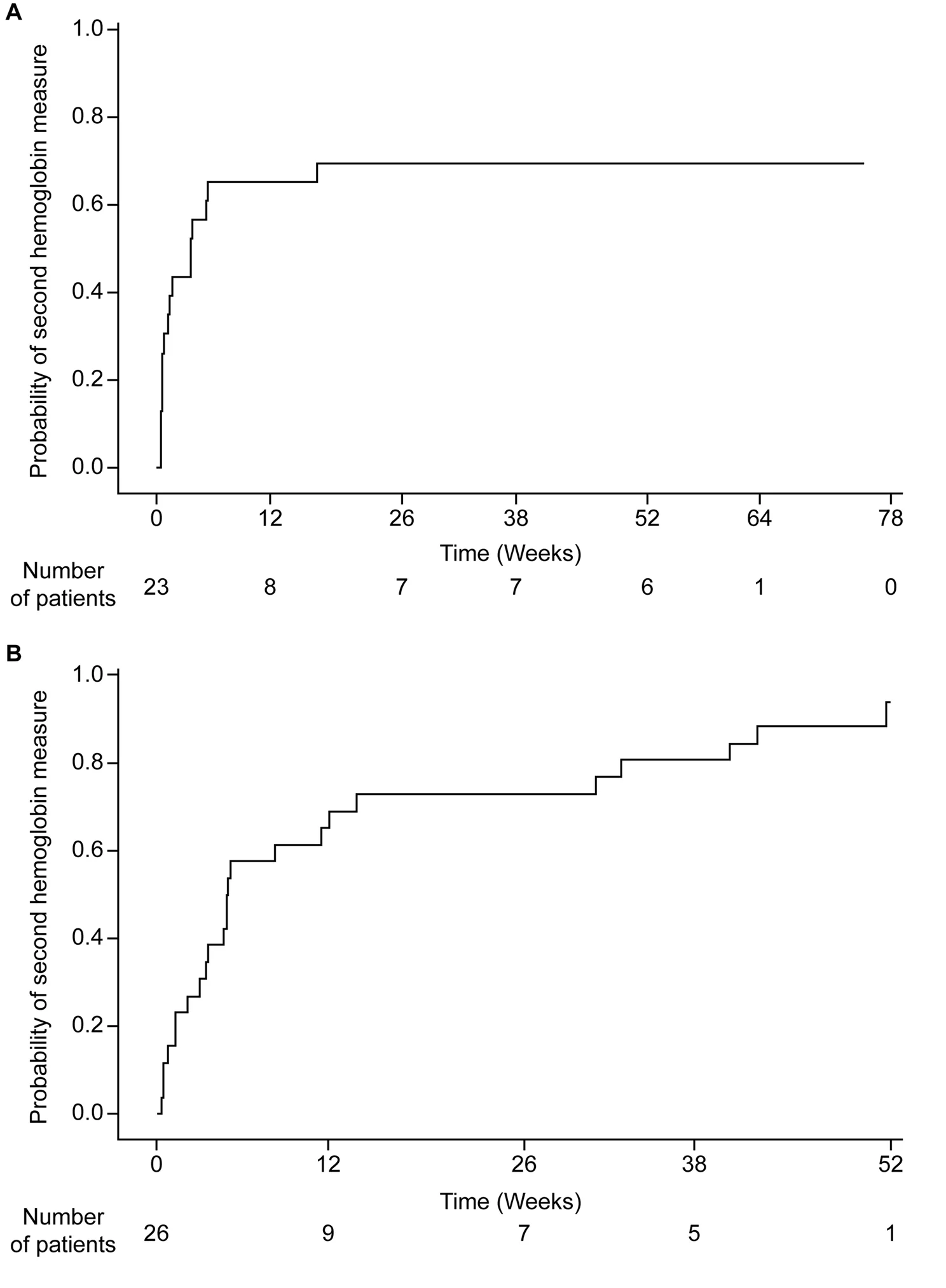
Time from index date to second hemoglobin measurement in the overall population according to dialysis status: (**A**) patients on dialysis at index date (*n* = 23)* (**B**) patients not on dialysis at index date (*n* = 26). *The probability of a second hemoglobin measurement did not reach 1.0, the reasons for which were not discernable from the data available

The time between tests was numerically different between patients on dialysis (median [IQR] 2.6 [1.0–4.3] weeks) and those not on dialysis (5.9 [3.0–8.4] weeks), and between patients receiving (3.4 [1.9–4.1] weeks) or not receiving kidney transplants (4.0 [1.7–8.0]), but this was not statistically assessed. The cumulative incidence curves showing the probability of receiving a second hemoglobin measurement in patients on dialysis at index date and in patients not on dialysis at index date are shown in Fig. 3A and B, respectively. The overall interval between hemoglobin tests for patients with ≥2 measurements had a median (IQR) of 4.0 (1.7–7.3) weeks; 3.4 (1.9–4.1) for eight patients receiving transplants and 4.0 (1.7–8.0) for 42 patients not receiving kidney transplants. For patients with ≥2 measurements observed, those from the UK and US had a median (IQR) of 4.0 (1.3–6.3) and 30.0 (4.9–31.6), respectively.

### Occurrence of AEs

A total of 20 AEs were recorded in nine (18.0%) patients during study follow-up. All were serious AEs (SAEs; Table 4); none were fatal or led to ESA treatment discontinuation. AEs were not attributable to any treatment and no inference was sought. Most SAEs (19/20; 95.0%) were classified as such due to the need for hospitalization or prolongation of existing hospitalization. Of the 20 SAEs, five were recorded in three patients on dialysis and 15 were recorded in six patients not on dialysis. SAE incidence was 2.0 (95% CI, 1.0–3.8) per 10 patient-years. The mean (SD) number of SAEs for patients with ≥1 SAE was 2.2 (1.3). These values were 1.7 (1.2) and 2.5 (1.4) for patients on and not on dialysis, respectively.

**Table 4:** Summary of SAEs by dialysis and transplant status*

SAE by system organ class, n (%)		Dialysis status at index date		Kidney transplant status **(post-index period)**^**†**^	
	Overall (n = 20)	Not on dialysis (n = 15)	On dialysis (n = 5)	No transplant (n = 13)	Transplant (n = 7)
Gastrointestinal disorders					
Vomiting	3 (15.0)	3 (20.0)	0 (0.0)	3 (23.1)	0 (0.0)
Infections and infestations					
Lower respiratory tract infection	3 (15.0)	2 (13.3)	1 (20.0)	1 (7.7)	2 (28.6)
Peritonitis	1 (5.0)	1 (6.7)	0 (0.0)	1 (7.7)	0 (0.0)
Septic shock	1 (5.0)	1 (6.7)	0 (0.0)	1 (1.7)	0 (0.0)
Urinary tract infection	2 (10.0)	2 (13.3)	0 (0.0)	0 (0.0)	2 (28.6)
Viral infection	1 (5.0)	1 (6.7)	0 (0.0)	0 (0.0)	1 (14.3)
Streptococcal bacteremia	1 (5.0)	1 (6.7)	0 (0.0)	0 (0.0)	1 (14.3)
Device-related infection	3 (15.0)	0 (0.0)	3 (60.0)	3 (23.1)	0 (0.0)
Metabolism and nutritional disorders					
Dehydration	2 (10.0)	2 (13.3)	0 (0.0)	1 (7.7)	1 (14.3)
Fluid overload	1 (5.0)	0 (0.0)	1 (20.0)	1 (7.7)	0 (0.0)
Renal and urinary disorders					
Acute kidney injury	1 (5.0)	1 (6.7)	0 (0.0)	1 (7.7)	0 (0.0)
Vascular disorders					
Hypovolemic shock	1 (5.0)	1 (6.7)	0 (0.0)	1 (7.7)	0 (0.0)

*The number of SAEs may be greater than the total number of patients as each patient can experience > 1 SAE. The percentages are derived based on the total number of SAEs reported from index date for patients in the relevant subgroup

^†^For patients without a kidney transplant, post-index follow-up period was a maximum of 52 weeks. For patients with a kidney transplant, post-index follow-up period could be up to a maximum of 104 weeks

## Discussion

This retrospective chart review was conducted to assess the feasibility of collecting accurate data related to patient and treatment characteristics, availability of hemoglobin data, and AEs from a sample of 50 pediatric patients receiving ESA treatment for anemia of CKD in the UK and US. The information presented here helps to improve the understanding of the representative granularity of routinely collected healthcare data relating to anemia of CKD and potential between-country differences. It may also assist in informing the design of future observational or interventional studies that seek to utilize routinely collected health records data.

Overall, data collected retrospectively from medical charts were largely available to inform on patient characteristics, disease management and treatment patterns. At the index date, datasets were mostly complete for hemoglobin measurements, CKD stage and ESA initial dose by line of treatment; dataset completeness was also high for the date of kidney transplant. The overall level and quality of data retrieved through the chart review methodology was better than had been anticipated, supporting the feasibility of using such methodology to inform the design of an external control arm for a single-arm clinical trial.

Through collecting data from UK- and US-based populations, we sought to gain an insight into the differences in data availability and healthcare practices of the two countries. There were substantial differences between the UK and US data availability and completeness of CKD diagnosis, date of renal anemia diagnosis and hemoglobin measurements, with availability and completeness being higher and missingness being lower in the UK. This may suggest variations in data collection methodology, data recording methods, and data retrievability practices across the UK and US. However, due to differences in the training and methodology used across the two countries and the small sample size of this study (only three UK centers and two US treating physicians participated in the study), further studies with a larger sample size are required to validate country-level observations.

The clinical monitoring of these patients included, as expected, frequent hemoglobin testing; overall, patients receiving dialysis had a median of 21.9 hemoglobin determinations during the first index-line ESA, with intervals between tests ranging from 0.1–17.4 weeks. There was high variation in hemoglobin testing, with differences apparent for all patient subgroups analyzed, but were most pronounced when comparing transplanted patients (within the eligibility period; *n* = 8) vs. non-transplanted patients (mean 57.9 vs. 14.5 tests) and patients receiving vs. not receiving dialysis (mean 31.4 vs. 13.6 tests). These differences likely reflect the requirements for routine monitoring of laboratory values for clinical care delivery and the varying ease in blood sampling for different patient groups, respectively. The variability in time between tests for patients with ≥2 hemoglobin tests observed for UK vs. US patients (mean 4.0 vs. 30.0 weeks, respectively) may be due to geographic variations in laboratory testing, for example, hemoglobin measurement and data recording in non-central laboratories which are a distance from transplantation centers in the US, vs. more local and/or harmonized data collection in UK. It may also reflect differences in medical record-keeping practices and feasibility of data retrieval. Furthermore, 7/8 patients with a kidney transplant during follow-up were in the UK; and receiving a transplant had a large effect on the number and frequency of hemoglobin tests in this study, in part due to their longer follow-up time (median [IQR] follow-up, 59.1 [41.1–80.0] transplanted [*n* = 8] vs. 52.1 [52.1–52.1] with no transplant [*n* = 42] weeks).

In this study, only 50% of patients who had a kidney transplant discontinued ESA therapy. Post-transplant anemia (PTA) is a common complication of kidney transplantation, with incidences ranging from ~30–50% [24–26]. Impaired kidney graft function, immunosuppressive therapy and transplant rejection can cause delayed kidney function, potentially leading to PTA [27]. In order to supplement erythropoietin production and reduce the risk of PTA, ESA therapy may be continued post-transplantation [28].

The frequency and range of comorbidities identified here (Supplementary Table 3) and in other studies [29–31] highlight the concurrent clinical disease burden within the pediatric population with anemia of CKD. Collection of data on comorbidities from medical charts could potentially aid identification of important contributors to the overall management needs and anemia risks of pediatric patients with CKD. For example, comorbidities observed in this study included hypertension, neurocognitive impairment and growth which could be important considerations based on their frequency, as observed in both adults and pediatric patients [23, 32]. These data reinforce existing records on the high comorbidity burden in pediatric CKD as a result of the combined effects of congenital, acquired, and non-reversible factors, and highlight the potential benefits of effective treatments for anemia of CKD [29–31].

The rate of AEs recorded in this retrospective chart review study was lower than reported in previous prospective studies [29–31, 33], which is likely due to AE under-recording and is compatible with only serious AEs being recorded and identifiable in the medical records. For comparison, a clinical trial by Schaefer et al. (2016) reported that over 50% of 319 pediatric patients with CKD experienced SAEs, over a median follow up of 88 weeks [29]. One reason for potential under-recording of AEs in this retrospective study in comparison to clinical trials may be due to deciding at what threshold a condition (e.g., hypertension) is a comorbidity or an AE. There was also a difference between the number of AEs reported by country, which may be a result of the site-based approach adopted in the UK vs. the panel-based approach used in the US (e.g., data access and extraction limitations, clinical coding, etc.). Furthermore, the SAEs observed in this study were considered to be consistent with those expected in a pediatric CKD and dialysis population. Their occurrence is therefore more likely reflective of the underlying disease burden, management, and treatment setting rather than ESA-related adverse effects [29, 33]. A formal attribution process was not undertaken during primary data collection. Future work should improve data collection by adopting the use of pre-specified AE lists as well as the option to enter free text to ensure that more AEs are captured. It may also be helpful to ask a separate question to assess whether the AE recorded is serious or not. Furthermore, data extraction and adjudication of AEs and SAEs by a second abstractor may be useful.

Strengths of this study comprise the inclusion of patients receiving and not receiving dialysis, the follow-up after renal transplantation, the capturing of CKD stage and dialysis transitions, as well as the inclusion of different types of healthcare settings in the UK and US. The importance of registry and cohort studies in the care of patients with CKD is well documented as they are key to informing and improving clinical practice through long-term observation of patients in a real-world setting. Previous cohort studies and registries have provided epidemiologic information as well as demonstrating the risk factors for mortality associated with pediatric patients with CKD [8]. Our study provided useful insights into the feasibility of retrospectively collecting medical chart review data which could be used in future studies to inform clinical knowledge and practice.

Limitations of this study include those associated with retrospective medical record reviews including reliance on the accuracy of data previously gathered for other purposes, the probability of missing data and the potential non-applicability of the data gathered to wider populations of patients and physicians.

Treating physicians were individually responsible for collecting patient data and were not given instructions on how to define CKD, raising the possibility of differences in the assessment and judgement of CKD stage between sites. Any patient without an eGFR reading may have been less likely to have a CKD stage assessment recorded. The request to collect eGFR data and CKD stage data in all participants, including patients receiving dialysis, did not account for the dialysis vs. non-dialysis population. Furthermore, the timings of laboratory measurements at the index date were not recorded. Future work is required to record the point at which laboratory values were taken. In addition, hemoglobin levels were not assessed by CKD stage at the index date. Therefore, determining whether patients with CKD stage 3 did or did not have anemia was not possible. Future work should assess the anemia status of patients relative to their CKD stage.

The objective of this study was to assess the feasibility of retrospectively collecting medical chart review data to use as an external control arm and to inform the design of future studies. Therefore, these data were not reported or analyzed further than general descriptions. Future studies are required to investigate treatment response to better understand factors which may be associated with treatment hyporesponsiveness in current clinical practice, and to analyze data longitudinally.

Potential parameters to address and assessments to make in future studies include the correlation between ESA type and anemia frequency and the effect of ESA treatment on anemia in real-world practice. These results confirmed the availability of the primary data elements required to assess such outcomes, demonstrating the feasibility of using retrospective chart review methodology in future studies. Whilst ESA dose relative to body surface area (BSA) is a reliable way to present ESA-dosing in pediatric renal anemia research due to the wide variation in BSA seen in populations aged < 18 years, it was not assessed in this study. This was due to missing height and weight data. Future studies are required to assess such parameters through the timed collection of height and weight data. Furthermore, collection of hematinic parameters data (e.g., baseline and longitudinal iron status, folate levels, vitamin B12 levels, or zinc protoporphyrin levels) and C-reactive protein levels, and assessments of ESA hyporesponsiveness should be undertaken in future studies.

All patients (incident and prevalent) were included in time-to-ESA discontinuation and time-to-ESA dose escalation calculations, including those with index dates prior to the eligibility period. Sub-group analyses were not undertaken to assess time-to-event outcomes in this study due to the small sample size. Therefore, sub-group analyses and future studies are required to assess time-to-event outcomes further.

Other potential limitations are linked to the inclusion of transplant patients, who were included in this study to track longitudinal transition state changes in hemoglobin levels, and ESA and iron use, and maintain cohort sample size over time; however, this may have affected hemoglobin measurement frequencies due to increased measurement (e.g. daily) in those who were recently transplanted. Most transplants occurred towards the later period in the standard 52-week follow-up course (median time to first kidney transplant from index date: 37.1 weeks). This did not prevent data interpretation for the study as designed and shown through the three subgroup analyses: (i) patients receiving vs. not receiving dialysis, (ii) transplanted and non-transplanted patients, and (iii) those from UK vs. US.

There was also potential for selection bias of patients by physicians, as well as information bias for pre-index period data, since only information accessible by sites/physicians in the patient’s medical records was reported. Some differences between data obtained for UK vs. US patients may be due to the provision of specific chart review training within the study. Among the numerous components in chart review studies that can introduce bias (e.g., poor data abstraction methodology), items outside the researcher’s control, such as incomplete, conflicting, or inaccurate data, may lead to incorrect conclusions. Although most pediatric nephrology clinics have electronic medical records, some do not and this could have had an impact on data availability. In this study, data availability was different between the UK and US, particularly regarding date of CKD or kidney anemia diagnosis, and hemoglobin measurements within pre-specified assessment windows. This assessment was included to help inform the absolute and relative data availability in electronic data systems for the outcomes being collected, as little existing qualitative information of this type was available in support of the initial study design. There may be opportunities for clinical data collection education in support of anemia of CKD surveillance, timely diagnosis, and implementation of management.

## Conclusions

This study demonstrates the feasibility of identifying clinical characteristics, treatments, and AEs from the medical charts of pediatric patients with anemia of CKD. Baseline hemoglobin levels, ESA treatment patterns, and frequency of iron treatment in incident and prevalent ESA-treated pediatric patients, both receiving and not receiving dialysis, provide key insights into current clinical management of anemia of CKD in pediatric patients. Such data could be used to inform the design of future similar studies, potentially supporting approaches to develop an external control arm in single-arm pediatric CKD clinical trials.

## Electronic supplementary material

Below is the link to the electronic supplementary material.

Supplementary Material 1

## Data Availability

For requests for access to anonymized subject level data, please contact the corresponding author (Anna Richards; http://anna.x.richards@gsk.com).
