# Supplementary material for: Characterization of pediatric patients receiving erythropoiesis-stimulating agents for management of anemia of chronic kidney disease in the UK and US: a feasibility assessment using retrospective chart review
Source: BMC Nephrol. 2026 Aug 27;27:532. doi: 10.1186/s12882-026-05188-2 (PMC13560262; doi:10.1186/s12882-026-05188-2)
Supplement: Supplementary file 1 [file 12882_2026_5188_MOESM1_ESM.docx]

**Supplementary Materials**

**Characterization of pediatric patients receiving erythropoiesis-stimulating agents for management of anemia of chronic kidney disease in the UK and US: a feasibility assessment of retrospective chart review study**

Sally Mountcastle, Keele Wurst, Alice Rouleau,^2^ Neil R Brett, Jennifer McKenzie, Justyna Amelio, Kathy Fraeman, Jake Hunnicutt, Susanna Cuadripani, David Webb, Amrit Kaur, Rukshana Shroff, Eleonora Staines Urias, Anna Richards,
Thomas F Hiemstra

**Supplementary Table 1** Feasibility assessment describing the missing medical history information for the study population by post-index date kidney transplant subgroups

| **Missing medical history information, *n* (%)** | **Overall (*N* = 50)** | **No transplant  (*n* = 42)** | **Post-index date transplant(s)  (*n* = 8)** |
| --- | --- | --- | --- |
| Date of initial CKD diagnosis | 7 (14.0) | 6 (14.3) | 1 (12.5) |
| CKD stage | 1 (2.0) | 1 (2.4) | 0 |
| eGFR | 15 (30.0) | 13 (31.0) | 2 (25.0) |
| Date of kidney anemia diagnosis | 14 (28.0) | 12 (28.6) | 2 (25.0) |
| Hemoglobin concentration | 1 (2.0) | 1 (2.4) | 0 |
| Date of hemoglobin measurement | 8 (16.0) | 7 (16.7) | 1 (12.5) |

CKD, chronic kidney disease; eGFR, estimated glomerular filtration rate.

**Supplementary Table 2** Feasibility assessment describing the missing medical history information for the study population by country subgroups

| **Missing medical history information, *n* (%)** | **Overall  (*N* = 50)** | **UK**  **(*n* = 36)** | **US**  **(*n* = 14)** |
| --- | --- | --- | --- |
| Date of initial CKD diagnosis | 7 (14.0) | 0 | 7 (50.0) |
| Date of kidney anemia diagnosis | 14 (28.0) | 6 (16.7) | 8 (57.1) |
| Date of kidney transplant (for those with previous transplant) | 0 | 0 | 0 |
| CKD stage | 1 (2.0) | 1 (2.8) | 0 |
| eGFR | 15 (30.0) | 7 (19.4) | 8 (57.1) |
| Hemoglobin concentration | 1 (2.0) | 1 (2.8) | 0 |
| Date of hemoglobin measurement | 8 (16.0) | 1 (2.8) | 7 (50.0) |

CKD, chronic kidney disease; eGFR, estimated glomerular filtration rate; UK, United Kingdom; US, United States.

**Supplementary Table 3** Comorbidities in dialysis and non-dialysis patients at the index date

| **Comorbidity, n (%)** | **Dialysis status at index** | | |
| --- | --- | --- | --- |
|  | **All**  **(*N* = 50)** | **Not on dialysis**  **(*n* = 27)** | **On dialysis**  **(*n* = 23)** |
| Hypertension | 15 (30.0) | 7 (25.9) | 8 (34.8) |
| Neurocognitive impairment | 11 (22.0) | 7 (25.9) | 4 (17.4) |
| Growth abnormalities | 10 (20.0) | 4 (14.8) | 6 (26.1) |
| Motor abnormalities | 8 (16.0) | 7 (25.9) | 1 (4.3) |
| Ocular abnormalities | 8 (16.0) | 4 (14.8) | 4 (17.4) |
| Pulmonary abnormalities | 8 (16.0) | 6 (22.2) | 2 (8.7) |
| Unknown | 8 (16.0) | 4 (14.8) | 4 (17.4) |
| Rare comorbidities* | 13 (26.0) | 10 (37.0) | 3 (13.0) |
| Other^†^ | 11 (22.0) | 4 (14.8) | 7 (30.4) |

*Specific comorbidities present at low numbers (*n* = 3 or less in the overall population) presented in aggregate to maintain anonymity

^†^Additional other comorbidities, nature unspecified.

**Supplementary Fig. 1** Time from index date to kidney transplant in the overall population (*N* = 50)

*Including all patients with available data who have not yet reached the outcome.
